# Targeting JAK2/STAT3-Dependent Macrophage Polarization by Chlorogenic Acid Attenuates Hepatic Inflammation in Chronic Stress

**DOI:** 10.3390/cells14231848

**Published:** 2025-11-24

**Authors:** Yaxin Ji, Haoyang Tan, Xin Cheng, Xiaoqing Yu, Jiahuan Hu, Jiaxing Wang, Haotian Yang, Guofeng Feng, Wenjing Jiao, Honggang Fan, Yuan Zhao

**Affiliations:** 1 Heilongjiang Key Laboratory for Laboratory Animals and Comparative Medicine, College of Veterinary Medicine, Northeast Agricultural University, Harbin 150030, China; 2 Heilongjiang Academy of Agricultural Science Branch of Animal Husbandry and Veterinary Branch, Qiqihar 161000, China

**Keywords:** chronic stress, CGA, liver inflammations, macrophage polarization, JAK2/STAT3 signaling pathway

## Abstract

**Highlights:**

**What are the main findings?**

- CGA alleviates chronic stress-induced liver injury and rebalances M1/M2 macrophage polarization, suppressing pro-inflammatory cytokines.
- CGA inhibits JAK2/STAT3 activation, validated by matching effects with the inhibitor S3I-201.

**What are the implications of the main findings?**

- CGA offers therapeutic potential for stress-related liver disorders via macrophage polarization modulation.

**Abstract:**

Chronic stress adversely affects and compromises physiological well-being in humans, inducing hepatic injury, with its pathogenesis mechanistically linked to alterations in macrophage polarization and the regulation of the inflammatory microenvironment. Chlorogenic acid (CGA), a principal active component of *Lonicera japonica* (honeysuckle), has been shown to have therapeutic effects on various liver diseases. However, the specific mechanism by which CGA confers hepatoprotective effects through the modulation of macrophage polarization and inflammatory responses remains unclear. In this study, rats were subjected to 6 h of daily restraint stress for 21 consecutive days, with the experimental group receiving concurrent administration of CGA (100 mg/kg, via gavage). The results demonstrated that CGA intervention effectively mitigated chronic stress-induced impairments in growth performance and hepatic structural and functional integrity. CGA significantly inhibited M1 macrophage polarization and the expression of pro-inflammatory cytokines (IL-6, IL-1β, and TNF-α), while simultaneously promoting M2 polarization and the expression of the anti-inflammatory cytokine IL-10. Furthermore, the administration of CGA was found to inhibit the activation of the JAK2/STAT3 signaling pathway. Additionally, the use of the JAK2/STAT3 signaling pathway inhibitor, S3I-201, demonstrated effects similar to those observed with CGA treatment. In summary, CGA modulates macrophage polarization and the inflammatory response through the regulation of the JAK2/STAT3 signaling pathway, thereby mitigating the liver injury induced by chronic stress.

## 1. Introduction

Modern life exposes individuals to persistent exogenous stimuli—including high-intensity workloads, social competition, and economic pressures—that potently trigger chronic stress responses. This pathological state significantly compromises psychological health, manifesting as reduced occupational and academic productivity, sleep disturbances, depressed mood, and elevated risks of anxiety disorders, major depressive disorder, and post-traumatic stress disorder (PTSD) [1,2]. Simultaneously, it inflicts severe physiological damage through impaired immune function, increased susceptibility to cardiovascular and digestive diseases, and heightened vulnerability to metabolic disorders, such as inflammatory bowel disease (IBD) and non-alcoholic fatty liver disease (NAFLD) [3,4]. Consequently, proactive implementation of chronic stress-mitigating interventions is essential for preserving psychosomatic health, enhancing quality of life, and preventing associated comorbidities.

As the central organ responsible for orchestrating metabolic regulation, immune modulation, and neuroendocrine integration, chronic stress-induced hepatic inflammation is recognized as a critical contributor to the pathogenesis and progression of chronic liver diseases, including hepatic fibrosis and hepatitis [5]. This stress-associated hepatic inflammation can further exacerbate systemic inflammatory responses, potentially leading to multi-organ damage [6,7]. Kronsten et al. revealed a mutually reinforcing pathological loop wherein depression accelerates liver disease (CLD) progression, while advancing liver dysfunction reciprocally worsens depressive symptoms [8]. Guo et al. demonstrated that hepatic inflammation serves as a critical driver in the initiation and progression of metabolic dysfunction-associated steatohepatitis (MASH) [9]. Adil Bhat et al. demonstrated in a chronic liver injury model that attenuating hepatic inflammation effectively ameliorates hepatic fibrosis [10]. A key mechanism underpinning hepatic inflammation involves dysregulation of macrophage polarization states [11]. Zibing Qian’s research highlights that the dynamic balance of macrophage M1/M2 polarization is critical for maintaining the homeostasis of the hepatic immune microenvironment and influencing disease progression [12]. Consequently, therapeutic strategies that focus on modulating macrophage polarization dynamics or the inflammatory microenvironment hold promise as pharmacological interventions for alleviating stress-induced hepatic injury.

Chlorogenic acid (CGA, C_16_H_18_O_9_), a naturally occurring phenolic compound, has demonstrated potent antioxidant, anti-inflammatory, and immunomodulatory properties. CGA effectively reduces the environmentally induced inflammatory responses [13]. Critically, CGA demonstrates significant anti-stress properties by modulating hypothalamic–pituitary–adrenal (HPA) axis hyperactivity and corticosterone (CORT) dysregulation [14]. Furthermore, its efficacy in ameliorating chronic hepatic disorders, such as cirrhosis, hepatitis, and NAFLD, is well documented [15,16]. The JAK2/STAT3 signaling pathway serves as a crucial regulatory hub for cytokine signal transduction within inflammatory networks. Mechanistic studies have demonstrated that the constitutive activation of this pathway is pathologically linked to various multiorgan inflammatory disorders [17,18,19,20]. In the hepatic context, this pathway promotes the polarization of Kupffer cells (KCs) towards pro-inflammatory M1 phenotypes, which is a critical pathogenic mechanism in the development of NASH [21]. Importantly, under chronic stress conditions, the JAK2/STAT3 signaling pathway mediates immunoregulatory dysfunction, contributing to an imbalance in cytokine secretion and macrophage polarization. This imbalance amplifies inflammatory cascades, ultimately resulting in the collapse of immune homeostasis and systemic inflammation [22]. Recent studies have shown that this signaling axis plays a pivotal role in governing macrophage phenotypic switching, thereby exerting significant regulatory control over the remodeling of the inflammatory microenvironment [23]. Research has shown that modulation of the JAK2/STAT3 signaling pathway is one mechanism by which CGA could achieve its anti-inflammatory effects. Xiaoying Yang demonstrated that CGA alleviates renal fibrosis by inhibiting JAK2/STAT3 signal transduction [24]; Qingqing Li further supported this mechanism in murine models of colitis, where CGA treatment resulted in suppressed STAT3 expression [25]. This inhibitory regulation of the JAK2/STAT3 pathway positions CGA as a critical modulator of inflammatory microenvironment remodeling. 

Building upon this evidence, this study investigates the role of CGA in modulating the JAK2/STAT3 signaling pathway and its downstream effects on macrophage polarization within the context of chronic stress-induced hepatic inflammation. Specifically, we aim to determine whether CGA attenuates chronic stress-induced hepatic injury by rebalancing macrophage polarization via inhibition of the JAK2/STAT3 pathway.

## 2. Materials and Methods

### 2.1. Animals 

Adult male Wistar rats weighing 200 ± 20 g were bought from Liaoning Changsheng Biotechnology Co., Ltd. (Shenyang, China). The rats were housed in a room that had a 12−12 h light/dark cycle (lights on from 6:00 to 18:00), with temperature and humidity maintained at 23 ± 2 °C and 45~55%, respectively, for 1 week to adapt to the environment. The rats were housed in groups (3 per cage) and had access to food and water ad libitum. All experimental procedures in this study met the requirements of the Animal Experimental Committee of Northeast Agricultural University (SRM11, China) and obeyed the National Institutes of Health Guide.

### 2.2. Treatment

Thirty-six male Wistar rats were randomly divided into six groups (*n* = 6): control (CON) group, chronic stress (CRS) group, chronic stress CGA-treated (CRS+CGA) group, control + inhibitor (CON + S3I-201) group, chronic stress + inhibitor (CRS + S3I-201) group, and chronic stress + inhibitor solvent (CRS+DMSO) group. Among them, rats in the CRS group, CRS + CGA group, CRS + S3I-201 group, and CRS + DMSO group were subjected to restraint stress for 6 h from 9:00 to 15:00 every day [26], and the rest of the groups of rats were fasted from food and water during this period, which lasted for 21 days. CGA (MB6178, Meilunbio, Dalian, China) was dissolved in distilled water (concentration: 12.5 mg/mL). The CRS + CGA group received CGA (100 mg/kg, gavage) at 8:00; the dosage of CGA was determined based on preliminary experimental studies [27]. S3I-201 (HY-15146, Med Chem Express, Shanghai, China) was dissolved in a mixed solution composed of 10% DMSO (472301, Sigma-Aldrich, Germany), 40% PEG300 (202371, Sigma-Aldrich, Germany), 5% Tween80 (P1754, Sigma-Aldrich, Germany), and 45% saline; The CON + S3I-201 group and the CRS + S3I-201 group received S3I-201 (5 mg/kg, i.p.) once every 3 days at 8:00. The CRS + DMSO group was the vehicle (1.25 mL/kg, once every 3 days) of S3I-201 at 8:00. Daily food intake was recorded and rats were weighed every three days during the modeling period. All rats were treated by anesthesia with isoflurane (Yipin Pharmaceutical Co., Ltd., Shijiazhuang, China). At 23 days, the blood and liver specimens were collected. All rats were alive until sacrificed.

### 2.3. Open Field Test (OFT)

All rats were subjected to the OFT after the model was established at 21 d. Before the test, a dim environment was maintained, and the rats were placed in the test room for acclimatization. At the beginning of the test, the rats were placed in the center of the experimental box (100 cm × 100 cm × 40 cm), and the test time for a single rat was 3 min. The behavior of rats (the crossing number, rearing number, and total distance of movement) was recorded by Supermaze software (V2.1, Shanghai XinRuan Information Technology Co., Ltd., Shanghai, China).27 After each test, the box was cleaned and dried thoroughly using 75% ethanol for the next test.

### 2.4. Enzyme-Linked Immunosorbent Assay (ELISA)

Blood was collected from the right ventricle of the rats’ hearts and centrifuged (1710· *g*, 10 min, 4 °C); after 30 min of clotting at 4 °C, serum was collected. Serum levels of CORT (H205) and inflammatory cytokines (IL-6: H007, IL-1β: H002, TNF-α: H052, IL-10: H009) were quantified using ELISA kits purchased from Nanjing Jiancheng Bioengineering Institute (Nanjing, China).

### 2.5. Serum ALT and AST Level Measurement 

Alanine aminotransferase (ALT, C009) and aspartate aminotransferase (AST, C010) serum levels were measured. The kits were obtained from Nanjing Jiancheng Bioengineering Institute (Nanjing, China).

### 2.6. Histological and Ultrastructural Observations

The liver tissues were embedded in paraffin after being fixed for 24 h. Sections were stained with hematoxylin and eosin (Wuhan Biotechnology Ltd. Co., Wuhan, China). After initial fixation overnight with 3% glutaraldehyde, the samples were washed three times with 0.1 M PBS buffer, each for 15 min, followed by postfixation with 1% osmium tetroxide solution for 2 h. After gradient dehydration, the tissues were embedded in a new type of epoxy resin, Epon, and polymerized at 60 °C for 2 h. Ultra-thin sections (60 nm) were double-stained with uranyl acetate and lead citrate. Observations were performed using a transmission electron microscope (Tecnai, Hitachi, Tokyo, Japan).

### 2.7. Immunohistochemistry (IHC) 

Paraffin sections were deparaffinized with xylene and dehydrated with gradient alcohol, antigen repaired by microwave, rinsed with PBS, and endogenous enzymes and non-specific sites were closed with 3% H_2_O_2_ and BSA. Primary antibody was incubated in a wet box at 4 °C overnight, and HRP secondary antibody was incubated at room temperature for 50 min. Primary antibodies for IHC were IL-6 (1:100, WL02841), IL-1β (1:100, WL02257), TNF-α (1:100, WL01581) and IL-10 (1:100, WL03088). DAB was visualized and monitored under microscope, and sections were transparent with hematoxylin staining followed by gradient dehydration, and neutral resin was used for microscopic examination. Sections were observed by microscope (Olympus, Tokyo, Japan). 

### 2.8. RT-PCR Analysis

The target genes were searched on the NCBI website, and primers were designed using Primer 5.0 and the sequences of the designed primers are shown in Table 1. The RNA of the liver was extracted according to the instructions of the total RNA extraction kit (Promega Biotech Co, Ltd., China), and the cDNA was obtained by reverse transcribing the total RNA using the GoScript Reverse Transcription System (Promega Biotech Co., Ltd., Beijing, China) [28]. RT-PCR was performed on a Light Cycler 480 II detection system (Roche, Basel, Switzerland).

### 2.9. Western Blot (WB)

The lysate (RIPA:PMSF:phosphatase inhibitor ratio of 98:1:1) was configured according to the 100-fold volume of the liver tissue, ground using a tissue grinder, and then centrifuged at 1710· *g* for 10 min at 4 °C. The supernatant obtained was the desired protein. Protein concentration was determined using a BCA Assay Kit (BCA Protein Assay Kit, Beyotime, Shanghai, China) and concentrated to 4 μg/μL by adding 0.9% NaCl solution and 5× sampling buffer, boiled for 10 min, and stored at −80 °C.

IL-6 (1:1000, WL02841), IL-1β (1:500, WL02257), TNF-α (1:1000, WL01581), IL-10 (1:1000, WL03088), JAK2 (1:500, WL02188), iNOS (1:500, WL0992a), Arg-1 (1:500, WL02825), and TGF-β (1:1000, WL02193) antibodies were purchased from Shenyang Wanlei Bio Technology Co., Ltd. (Shenyang, China).; STAT3, p-STAT3, p-JAK2 antibodies were purchased from Cell Signaling Technology (Danvers, MA, USA); CD86 (1:1000, #DF6332) was purchased from Affinity Biosciences (Cincinnati, OH, USA). The membranes were washed four times with TBST. The membranes were then incubated with horseradish peroxidase-conjugated goat anti-rabbit IgG antibody (ZB-2301, 1:20,000, ZSGB-BIO) for 2 h at room temperature and then washed five times with TBST for 5 min each. The ECL plus detection system (Tanon 5200, Shanghai, China) was used to visualize membranes. Then, the protein gray value was detected by ImageJ (1.54 f) software.

### 2.10. Immunofluorescence (IF)

For immunofluorescence single-staining detection of intracellular phosphorylated protein p-STAT3, cells were fixed (4% paraformaldehyde), membrane-broken (0.1% Triton X-100), closed and sequentially incubated with p-STAT3 primary antibody (1:500, #9139) and fluorescent secondary antibody, and nuclei were stained with DAPI. When double-staining for the detection of surface antigens CD86 (1:200, WL05184) and F4/80 (1:500, GB113373), mouse anti-CD86 and rabbit anti-F4/80 primary antibodies were mixed directly after fixation, and species-specific fluorescent secondary antibodies were added after incubation, protected from light to distinguish the signals through different excitation channels and to avoid cross-reactivity.

### 2.11. Statistical Analysis

All statistical analyses were performed using Grap CORT d Prism 9.5 software. Data are expressed as mean ± standard deviation (Mean ± SD). Intergroup comparisons were analyzed by Student’s *t*-test, with *p* < 0.05 considered statistically significant and *p* < 0.01 indicating highly significant differences.

## 3. Results

### 3.1. Validation of the Chronic Stress Model 

**Figure 1:**
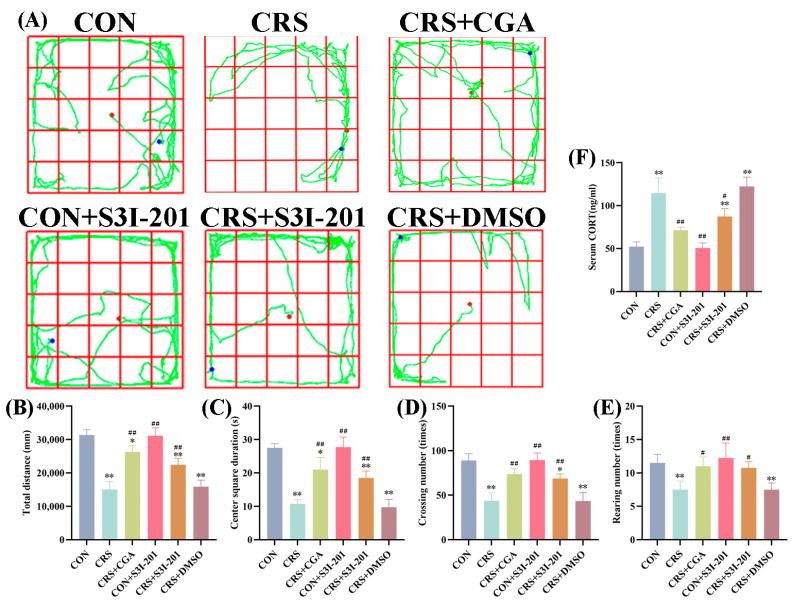
Validation of the chronic stress model (*n* = 3). Adult male Wistar rats were subjected to daily 6 h restraint stress (CRS) for 21 days or left unstressed (CON). Treatment groups received chlorogenic acid (CGA, 100 mg/kg/day, gavage), the JAK2/STAT3 inhibitor S3I-201 (5 mg/kg, i.p., every 3 days), or vehicle (DMSO, i.p., every 3 days) as indicated. (**A**). The route of the open field test in rats; (**B**). the total distance of the open field test in rats; (**C**). the center square of the open field test in rats; (**D**). the crossing number of the open field test in rats; (**E**). the rearing number of the open field test in rats; (**F**). the serum CORT content in rats. “*” indicates a significant difference compared with the CON group, *p* < 0.05. “**” indicates a highly significant difference compared with the CON group, *p* < 0.01. “#” means a significant difference compared with the CRS group, *p* < 0.05. “##” means an extremely significant difference compared with the CRS group, *p* < 0.01.

Validation of the chronic stress model in rats, along with the effects of different interventions, is depicted in Figure 1A–F. The model was first validated by comparing the CON group with the CRS group; consistent with the CON group, the CRS group showed a significant reduction in total distance traveled (Figure 1B), center square duration (Figure 1C), crossing number (Figure 1D), and rearing number (Figure 1E) in the open field test (*p* < 0.01), while serum CORT levels were significantly elevated (Figure 1F, *p* < 0.01). These results confirm the successful establishment of the chronic stress model.

Notably, the CRS + CGA group partially reversed the CRS-induced behavioral deficits; compared to the CRS group, CRS + CGA rats showed significantly increased total distance, center square duration, crossing number, and rearing number (*p* < 0.01), accompanied by a marked reduction in serum CORT levels (*p* < 0.01). This suggests that CGA may mitigate chronic stress-induced abnormalities. For the S31-201 intervention, the CRS + S31-201 group showed significantly improved behavioral or physiological outcomes compared to CRS (*p* > 0.05), while the CON + S31-201 group showed no significant differences from the CON group (*p* > 0.05), indicating S31-201 alone does not affect normal rats. The CRS + DMSO group, as a solvent control, exhibited results nearly identical to the CRS group (*p* > 0.05), confirming DMSO did not interfere with the stress response.

These findings collectively validate the chronic stress model and provide preliminary evidence for the differential effects of CGA and S31-201 on stress-induced behavioral and physiological changes.

### 3.2. Effects of Body Weight and Serum Inflammatory Cytokine Changes 

**Figure 2:**
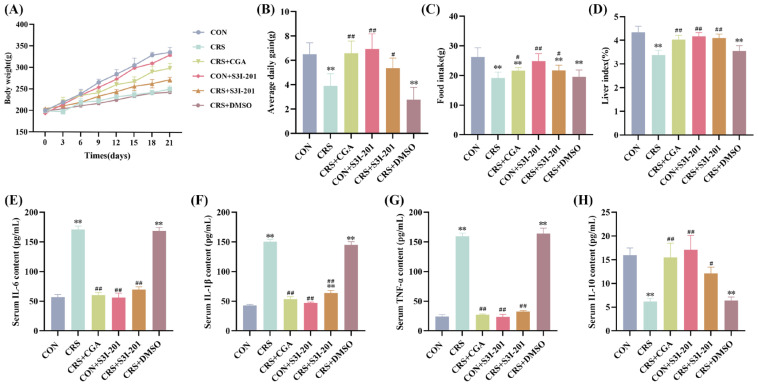
Results of changes in body weight and serum inflammatory cytokines in rats (*n* = 3). Rats were treated as described in Figure 1 legend. (**A**). Change in rat body weight; (**B**). average daily weight gain in rats; (**C**). changes in daily feed intake in rats; (**D**). liver coefficient in rats; (**E**–**H**). analysis of IL-6, IL-1β, TNF-α, and IL-10 serum ELISA results, respectively. “**” indicates highly significant difference compared with CON group, *p* < 0.01. “#” means a significant difference compared with CRS group, *p* < 0.05. “##” means an extremely significant difference compared with CRS group, *p* < 0.01.

Changes in body weight and daily feed intake of rats are shown in Figure 2A–D. Throughout the experimental period, all groups exhibited weight gain; however, rats in the CRS group experienced a slight body weight reduction on day 3, whereas the remaining groups displayed continuous increases. Compared with the CON group, both the CRS and CRS + DMSO groups showed significantly slower weight gain (Figure 2A), reduced average daily weight gain (Figure 2B), decreased daily feed intake (Figure 2C), and diminished liver index (Figure 2D) (*p* < 0.01). Compared with the CRS group, the CON + CGA and CRS + S3I-201 groups exhibited varying degrees of improvement in these parameters (*p* < 0.05).

ELISA results for serum inflammatory cytokines are presented in Figure 2E–H. Compared with the CON group, the serum IL-6, IL-1β and TNF-α levels of rats in the CRS group and the CRS + DMSO group were all extremely significant increases, and IL-10 was extremely significant decreases (*p* < 0.01); compared with the CRS group, the serum inflammatory cytokine levels of rats in the CRS + CGA group, the CON + S3I-201 group, and the CRS + S3I-201 group were all significantly restored (*p* < 0.05). 

Collectively, these findings indicate that chronic stress markedly suppressed growth and appetite and induced a systemic inflammatory response in rats, while both CGA and S3I-201 treatments effectively mitigated these adverse effects.

### 3.3. Hepatic Structural and Functional Alterations 

**Figure 3:**
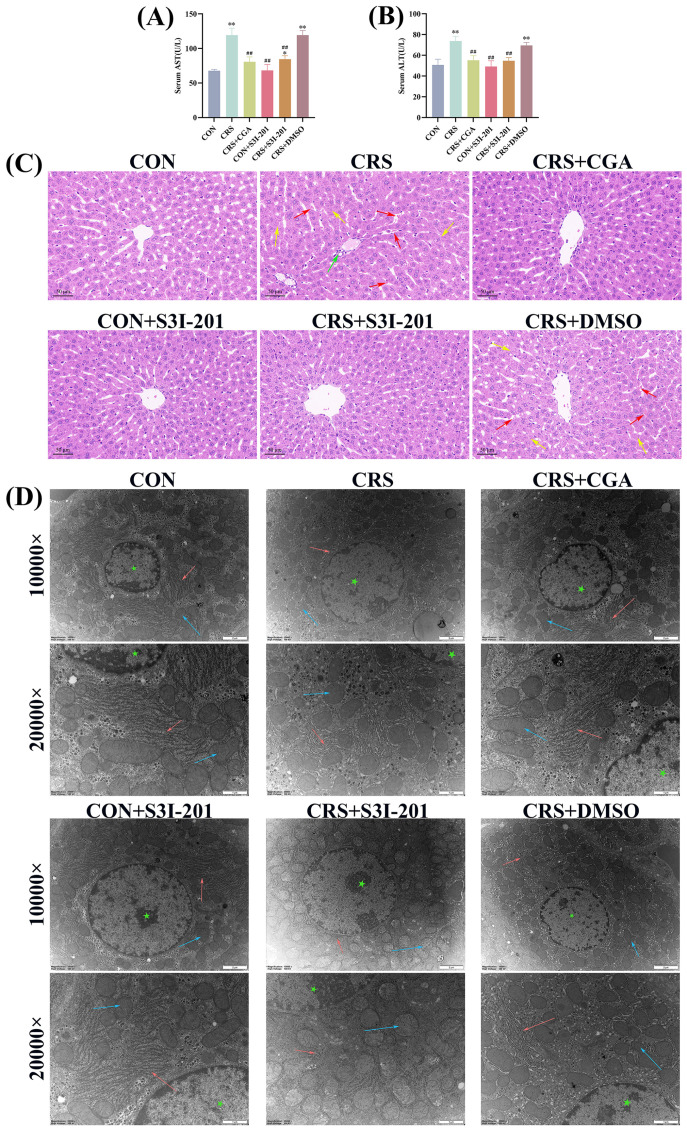
Structural and functional changes (*n* = 3)**.** Rats were treated as described in Figure 1 legend. (**A**,**B**). Results of hepatic function assay in rats’ liver; (**C**). histopathological changes in liver in rats (200×); (**D**). ultrastructural observations of liver in rats (10,000× and 20,000×). Figure 3C red arrows indicate bleeding; green arrow indicates inflammatory infiltration of cells; yellow arrows indicate cell necrosis; Figure 3D red arrows indicate endoplasmic reticulum; blue arrow indicates inflammatory infiltration of mitochondria; green asterisk indicates nucleus. “*” indicates significant difference compared with CON group, *p* < 0.05. “**” indicates highly significant difference compared with CON group, *p* < 0.01. “##” means extremely significant difference compared with CRS group, *p* < 0.01.

The results of the hepatic function assessment are depicted in Figure 3A,B. Compared with the CON group, serum AST and ALT levels were significantly elevated in both CRS and CRS + DMSO groups (*p* < 0.01). Relative to the CRS group, the CRS + CGA, CON + S3I-201, and CRS + S3I-201 groups exhibited significantly reduced serum AST and ALT levels (*p* < 0.01). 

Figure 3C presents the histopathological examination of rat liver tissue. The analysis indicated that the CON, CRS + CGA, CON + S3I-201, and CRS + S3I-201 groups maintained intact hepatic lobular architecture with well-defined boundaries, appropriately positioned central veins, orderly hepatocyte cord arrangements, and normal cellular morphology without signs of necrosis or hemorrhage. In contrast, the CRS and CRS + DMSO groups showed indistinct lobular demarcation, disorganized hepatocyte cords, sporadic hemorrhagic foci, and inflammatory cell infiltration, with additional inflammatory cellular infiltration observed specifically in the CRS group. The ultrastructural morphology of hepatocytes is illustrated in Figure 3D. The experimental groups, including CON, CRS + CGA, CON + S3I-201, and CRS + S3I-201, demonstrated preserved cellular architecture characterized by a widespread distribution of well-organized endoplasmic reticulum and mitochondria. In contrast, the CRS and CRS + DMSO groups exhibited disrupted organelle organization, notably with significantly swollen endoplasmic reticulum and diminished ribosome attachment, as well as mitochondria with blurred or absent cristae. These observations indicate that chronic stress precipitated both structural and functional hepatic damage, whereas CGA treatment effectively ameliorated such damage by inhibiting the JAK2/STAT3 signaling pathway.

### 3.4. Hepatic Macrophage Polarization Profiling

**Figure 4:**
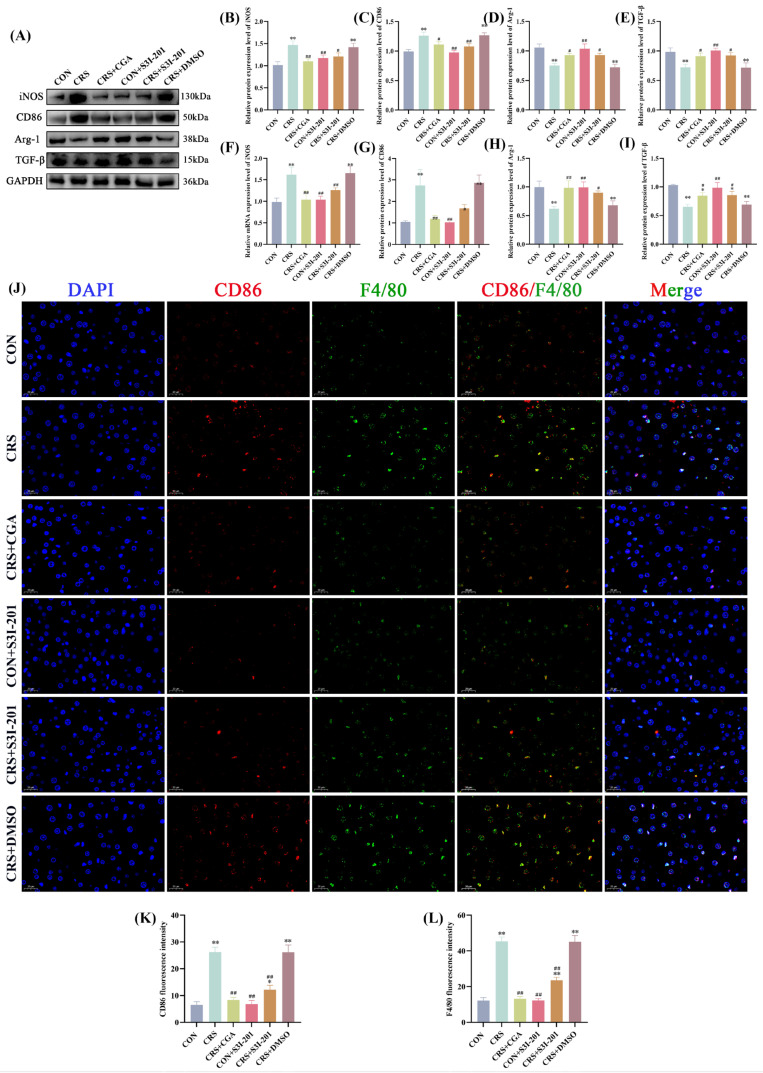
Results of macrophage polarization assay in rat livers (n = 3). Rats were treated as described in Figure 1 legend. (**A**). Graphs of Western Blot experiment results; (**B**–**E**). analysis of iNOS, CD86,Arg-1, and IL-10 protein expression results; (**F**–**I**). analysis of IL-6, IL-1β, TNF-α, and IL-10 gene expression data; (**J**–**L**). results and data analysis of immunodouble fluorescence staining of macrophage M1-type polarization markers. “*” indicates significant dqinghuidaifference compared with CON group, *p* < 0.05. “**” indicates highly significant difference compared with CON group, *p* < 0.01. “#” means significant difference compared with CRS group, *p* < 0.05. “##” means extremely significant difference compared with CRS group, *p* < 0.01.

Figure 4A–I present the protein and gene expression levels of macrophage polarization markers in rat livers. Compared to the CON group, the CRS and CRS + DMSO groups showed significantly increased protein and mRNA levels of M1 polarization markers (iNOS, CD86) and decreased levels of M2 markers (Arg-1, TGF-β) (*p* < 0.01). Conversely, the CRS + CGA, CON + S3I-201, and CRS + S3I-201 groups exhibited a differential restoration of polarization marker expression relative to the CRS group (*p* < 0.05). IF co-staining of the M1 marker CD86 with the pan-macrophage marker F4/80 is presented in Figure 4J–L. The fluorescence intensities of CD86 and F4/80 were significantly elevated in the CRS group, CRS + DMSO group, and CRS + S3I-201 group compared to the CON group (*p* < 0.05). Furthermore, the CRS + CGA group, CON + S3I-201 group, and CRS + S3I-201 group exhibited statistically significant reductions in fluorescence intensities relative to the CRS group (*p* < 0.01). 

These findings indicate that chronic stress enhances M1 polarization while inhibiting M2 polarization of hepatic macrophages, with both S3I-201 and CGA treatments effectively mitigating this imbalance.

### 3.5. Hepatic Inflammatory Cytokine Expression Profiles 

**Figure 5:**
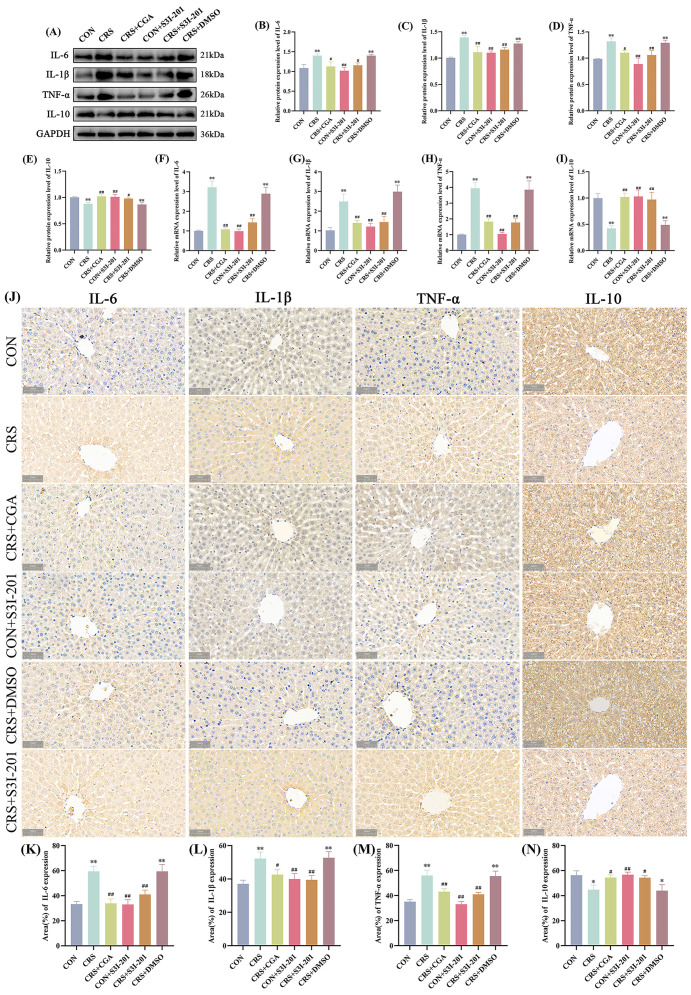
Results of inflammatory expression detection (*n* = 3). Rats were treated as described in Figure 1 legend. (**A**). Graphs of Western Blot experiment results; (**B**–**E**). analysis of IL-6, IL-1β, TNF-α, and IL-10 protein expression results; (**F**–**I**). analysis of IL-6, IL-1β, TNF-α, and IL-10 gene expression data; (**J**). graphs of immunohistochemical results; (**K**–**N**). analysis of IL-6, IL-1β, TNF-α, and IL-10 immunohistochemical results. “*” indicates significant difference compared with CON group, *p* < 0.05. “**” indicates highly significant difference compared with CON group, *p* < 0.01. “#” means significant difference compared with CRS group, *p* < 0.05. “##” means extremely significant difference compared with CRS group, *p* < 0.01.

The expression profiles of hepatic inflammatory cytokines are depicted in Figure 5. In comparison to the CON group, the CRS and CRS + DMSO groups showed significantly increased protein and gene expression levels of IL-6, IL-1β, and TNF-α (*p* < 0.01), along with significantly decreased IL-10 expression (*p* < 0.01). In contrast, the CRS + CGA, CON + S3I-201, and CRS + S3I-201 groups exhibited significantly reduced expression of IL-6, IL-1β, and TNF-α, accompanied by increased IL-10 levels relative to the CRS group (*p* < 0.01). The IHC findings were consistent. These results indicate that chronic stress disrupts inflammatory cytokine homeostasis to promote hepatic inflammatory responses, while both CGA and S3I-201 treatments effectively restore this balance.

### 3.6. Detection of JAK2/STAT3 Signaling Pathway in Rat Liver 

**Figure 6:**
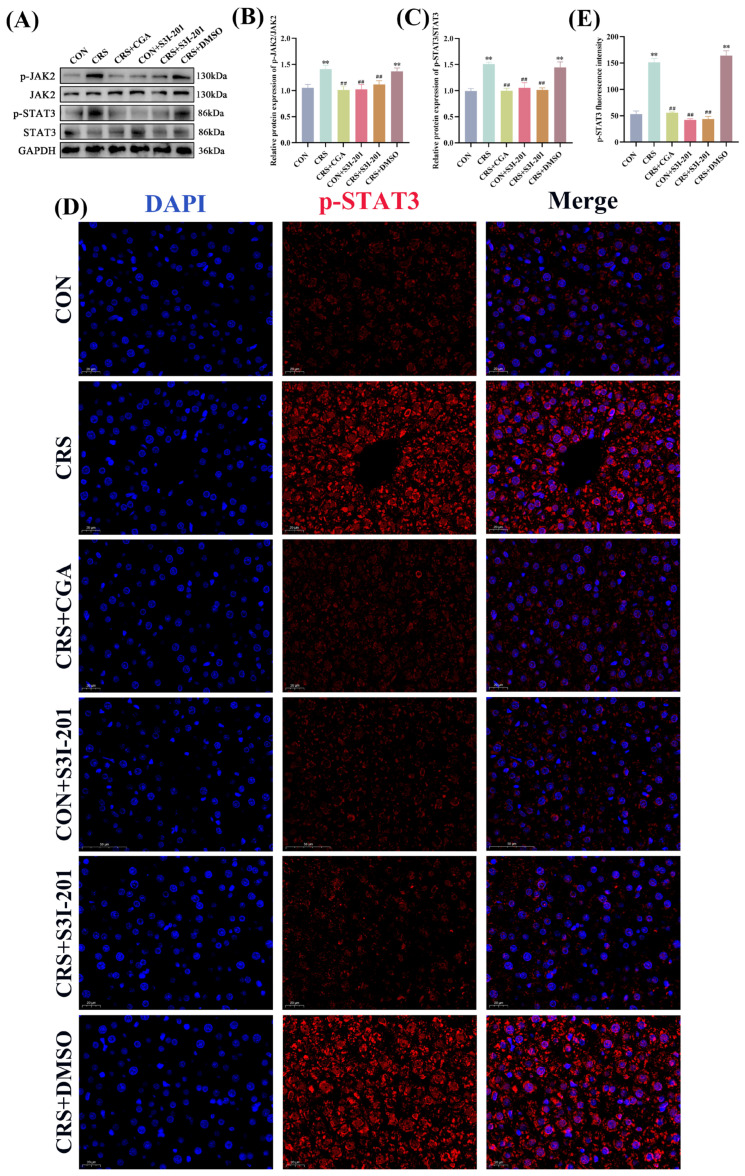
Results of JAK2/STAT3 signaling pathway detection (*n* = 3). Rats were treated as described in Figure 1 legend. (**A**–**C**). JAK2/STAT3 signaling pathway protein expression results and data analysis; (**D**,**E**). p-STAT3 immunofluorescence staining results and data analysis. “**” indicates highly significant difference compared with CON group, *p* < 0.01. “##” means extremely significant difference compared with CRS group, *p* < 0.01.

As a pivotal regulatory factor in cellular signal transduction, STAT3 predominantly resides in the cytoplasm in an inactivated form under quiescent conditions, with phosphorylated STAT3 translocating to the nucleus to mediate target gene transcription. The JAK2/STAT3 signaling pathway protein expression profiles are presented in Figure 6A–C. Compared with the CON group, the CRS and CRS + DMSO groups exhibited significantly elevated relative protein expression levels of p-JAK2 and p-STAT3 (*p* < 0.01). In contrast, the CRS + CGA, CON + S3I-201, and CRS + S3I-201 groups exhibited significantly reduced levels of p-JAK2 and p-STAT3 expression relative to the CRS group (*p* < 0.01). IF analysis of p-STAT3 nuclear translocation in liver tissue (Figure 6D,E) indicated lower levels of nuclear translocation in the CON, CRS + CGA, CON + S3I-201, and CRS + S3I-201 groups, whereas the CRS and CRS + DMSO groups showed increased nuclear accumulation. These findings collectively indicate that chronic stress robustly activates the JAK2/STAT3 signaling pathway, with both S3I-201 and CGA treatments exerting significant inhibitory effects.

## 4. Discussion

Chronic stress detrimentally affects human growth and physiological homeostasis. CGA, a principal component of Lonicera japonica, has demonstrated hepatoprotective efficacy in various liver disease models [29]. Our study revealed that CGA significantly suppresses the activation of the JAK2/STAT3 pathway. This study employed the JAK2/STAT3 signaling pathway inhibitor S3I-201 for mechanistic intervention, thereby definitively elucidating the cytoprotective mechanisms through which CGA attenuates chronic stress-induced hepatic injury.

To explore the mechanisms underlying chronic stress-induced injury and develop effective intervention strategies, the establishment of a reliable animal model is essential. This study utilized the chronic restraint stress (CRS) model, which is extensively validated for its non-invasive nature, operational simplicity, and high degree of standardization [30]. Evaluation metrics for chronic stress models primarily include behavioral assessments and hormone level measurements. The Open Field Test (OFT) is a widely employed experimental method in animal behavioral research due to its ease of operation, speed, and high efficiency. In OFT evaluations, the CRS rats demonstrated significantly reduced total distance traveled, shorter duration spent in the central area, fewer grid crossings, and decreased rearing frequency—behavioral indicators suggestive of heightened anxiety and stress levels [31]. Chronic stress activates the HPA axis, resulting in prolonged glucocorticoid release, which in turn leads to depressive-like behaviors in animals. CORT, the primary glucocorticoid secreted in rodents, serves as a direct indicator of systemic stress responses and is critically important for evaluating chronic stress models [32]. The serum CORT levels in CRS rats were significantly elevated. The integration of OFT behavioral assessments and serum CORT biochemical measurements effectively validated the establishment of a chronic stress animal model.

Monitoring essential physiological parameters, such as body weight and feed intake, is crucial for understanding growth patterns and evaluating health status. Our study found that chronic stress markedly decreased body weight and appetite in rat models, aligning with findings from various other chronic stress model systems [33,34]. Notably, stress-induced weight loss is directly associated with reduced feed conversion efficiency in livestock production. Moreover, perturbations in inflammatory cytokines serve as critical biomarkers for assessing immune homeostasis. Chronic stress exposure concurrently elevated serum levels of pro-inflammatory cytokines (IL-6, IL-1β, TNF-α) and depressed anti-inflammatory IL-10 expression. These findings suggest stress-induced immune dysregulation and the onset of systemic inflammation. This mechanistic alignment with Kandilarov’s findings [35] highlights a close association between chronic stress-induced damage and inflammatory responses. CGA demonstrates protective efficacy against chronic stress-induced hepatic injury. Based on preliminary laboratory dose–response studies, an optimal intervention dose of 100 mg/kg was determined [27]. The results demonstrated that CGA effectively reversed chronic stress-induced reductions in body weight and appetite, mitigated alterations in serum inflammatory cytokine levels, and ameliorated systemic inflammatory responses in rats.

The liver, as the primary organ for synthesis and metabolism, is often implicated in systemic growth performance impairment and the onset of inflammatory responses, which are closely linked to structural and functional hepatic damage, forming a critical pathophysiological basis for these manifestations. Serum AST and ALT are considered gold-standard biomarkers for hepatic functional assessment, with significantly elevated levels indicating liver injury. In CRS-exposed rats, there were marked elevations in serum AST and ALT activities; however, intervention with CGA significantly normalized these biochemical markers. Hepatic cords in CRS-exposed rats exhibited disorganized architecture with swollen mitochondria and endoplasmic reticulum within hepatocytes, indicating structural damage to the parenchyma. In contrast, rats treated with CGA exhibited well-preserved hepatic lobular architecture and intracellular ultrastructure, with no notable alterations in mitochondria or endoplasmic reticulum. These findings collectively underscore CGA’s ability to ameliorate histopathological alterations and mitigate hepatic parenchymal injury, thereby affirming its hepatoprotective efficacy against chronic stress-induced liver damage, in alignment with previous studies. Furthermore, given the liver’s critical role in immune homeostasis dysregulation, this study quantified fluctuations in inflammatory cytokines within hepatic tissues. This study evaluated inflammatory cytokine level alterations within hepatic tissues, demonstrating consistency with serum profile changes, thereby confirming localized inflammation occurrence in the liver parenchyma. This pathophysiological manifestation supports the foundational findings of Vlad Țâru et al. regarding chronic hepatic pathologies [36]. CGA mitigated hepatic inflammatory injury associated with chronic stress by downregulating the expression of pro-inflammatory cytokines (IL-6, IL-1β, and TNF-α) and upregulating the levels of the anti-inflammatory cytokine IL-10. These results are mechanistically consistent with the observations reported by Azam Moslehi et al. in murine models of endoplasmic reticulum stress [37].

Inflammatory responses are triggered by pathogen invasion, tissue injury, or disruptions in immune homeostasis, with macrophages being the predominant immune cell population in the liver. These cells play a crucial role in maintaining hepatic homeostasis and orchestrating the pathological mechanisms underlying liver diseases [21]. Critically, the initiation of hepatic inflammation is mechanistically entwined with alterations in macrophage polarization states. Macrophages originate as the M0 phenotype and undergo dichotomous polarization toward either the M1 or M2 phenotype contingent upon immunomodulatory microenvironmental cues. Empirical evidence confirms that M1 macrophages—activated by lipopolysaccharide (LPS) and analogous stimuli—exert pro-inflammatory effector functions characterized by surface markers (e.g., iNOS, CD86) and secrete cytokines, including IL-1β and TNF-α. Conversely, M2 macrophages polarize following TLR4-mediated activation, producing resolution mediators such as IL-10 and expressing surface markers (e.g., Arg1, CD206). Consequently, iNOS and CD86 were designated as M1 polarization markers, whereas Arg1 and CD206 served as M2 polarization indices. Notably, CRS elevated hepatic macrophage M1 polarization while suppressing M2 polarization, aligning with the pathognomonic shifts documented by Zahra Mirsanei et al. in LPS-driven inflammatory disease models [38]. Additionally, in alignment with Siyuan Xiong’s research [39], which demonstrated that CGA mitigates neuroinflammation-induced cognitive dysfunction through the inhibition of microglial M1 polarization, our study found that CGA administration reversed chronic stress-induced alterations in macrophage polarization states in the liver. Specifically, CGA treatment was associated with reduced M1 polarization and the reinstatement of M2 polarization. These results further support the notion that the anti-inflammatory effects of CGA are mechanistically linked to the restoration of hepatic macrophage polarization homeostasis. Nonetheless, the precise mechanisms underlying CGA’s protective role against chronic stress-related hepatic inflammation remain inadequately defined.

The JAK2/STAT3 signaling pathway is a pivotal component in the regulation of inflammatory responses [40]. CGA significantly suppressed aberrant activation of the JAK2/STAT3 signaling pathway, thereby further confirming its regulatory action on this pathway. To elucidate the cytoprotective mechanisms of CGA against hepatic inflammation induced by chronic stress, this study utilized the JAK2/STAT3 pathway inhibitor S3I-201 for targeted intervention in the cohort subjected to the CRS group. Post-intervention analyses revealed marked restoration of growth performance and hepatic functional-structural integrity, accompanied by reduced expression of M1 macrophage polarization markers (CD86, iNOS) and promotion of anti-inflammatory M2 phenotypes (Arg1, CD206). Concurrently, it reduced pro-inflammatory cytokine levels (IL-6, IL-1β, and TNF-α) while elevating anti-inflammatory IL-10 expression, thereby effectively ameliorating hepatic inflammatory injury. The findings collectively indicate that inhibition of the JAK2/STAT3 pathway mitigates stress-induced hepatic inflammatory injury by reprogramming macrophage polarization dynamics, aligning mechanistically with Jinmei Yao’s observations in non-alcoholic steatohepatitis models [41]. This suggests that JAK2/STAT3 pathway inhibition produces effects similar to those of CGA intervention, thereby unequivocally establishing that CGA confers hepatoprotection by suppressing aberrant activation of the JAK2/STAT3 signaling pathway. 

## 5. Conclusions

Collectively, this study demonstrates that CGA ameliorates chronic stress-induced hepatic injury in rats by suppressing JAK2/STAT3 pathway activation, which orchestrates a macrophage phenotypic reprogramming from pro-inflammatory M1 to anti-inflammatory M2 polarization. This shift attenuates pro-inflammatory cytokine secretion, restores intrahepatic immune homeostasis, and ultimately confers hepatoprotection through breaking the stress-initiated inflammatory cascade.

## Figures and Tables

**Table 1:** RT-PCR primer sequence.

Gene	Accession Number	Primer Sequence (5′−3′)
*GAPDH*	NM_017008.4	forward: AGTGCCAGCCTCGTCTCATA
		reverse: GATGGTGATGGGTTTCCCGT
*IL-6*	NM_012589.2	forward: AGAGACTTCCAGCCAGTTGC
		reverse: TGCCATTGCACAACTCTTTTC
*IL-1β*	NM_031512.2	forward: CAGCTTTCGACAGTGAGGAGA
		reverse: CTCCACGGGCAAGACATAGG
*TNF-α*	NM_012675.3	forward: ATGGGCTCCCTCTCATCAGTT
		reverse: GCTTGGTGGTTTGCTACGAC
*IL-10*	NM_012854.2	forward: AAGGGTTACTTGGGTTGCCA
		reverse: GGGAGAAATCGATGACAGCGT
*CD86*	NM_020081.3	forward: GATTGCAGGTCCCAGTTCACTTC
		reverse: CCACTGTCCTGCTTGGACTCAC
*iNOS*	NM_012611.4	forward: TTGGCTCCAGCATGTACCCT
		reverse: TCCTGCCCACTGAGTTCGTC
*Arg-1*	NM_017134.3	forward: CAGTATTCACCCCGGCTA
		reverse: CCTCTGGTGTCTTCCCAA
*TGF-β*	NM_021578.2	forward: CCAGATCCTGTCCAAACTAAGG
		reverse: CTCTTTAGCATAGTAGTCCGCT

## Data Availability

The data that support the findings of this study are available from the corresponding author upon reasonable request.

## Abbreviations

ALT	Alanine Aminotransferase
Arg1	Arginase-1
AST	Aspartate Aminotransferase
CD206	Cluster of Differentiation 206
CD86	Cluster of Differentiation 86
CGA	Chlorogenic Acid
CON	Control
CORT	Corticosterone
CRS	Chronic Restraint Stress
DAPI	Dihydrochloride
DMSO	Dimethyl Sulfoxide
ELISA	Enzyme-Linked Immunosorbent Assay
GAPDH	Glyceraldehyde-3-Phosphate Dehydrogenase
HPA	Hypothalamic–Pituitary–Adrenal
IBD	Inflammatory Bowel Disease
IF	Immunofluorescence
IHC	Immunohistochemistry
IL-10	Interleukin-10
IL-1β	Interleukin-1beta
IL-6	Interleukin-6
iNOS	Inducible Nitric Oxide Synthase
JAK2	Janus Kinase 2
KCs	Kupffer Cell
LPS	Lipopolysaccharide
NAFLD	Non-Alcoholic Fatty Liver Disease
NASH	Nonalcoholic Steatohepatitis
OFT	Open Field Test
SD	Standard Deviation
STAT3	Signal Transducer and Activator of Transcription 3
TBST	Tris-Buffered Saline with Tween 20
TLR4	Toll-Like Receptor 4
TNF-α	Tumor Necrosis Factor-A
WB	Western Blot
